# Thrombotischer Verschluss der extrakorporalen Zirkulation während hepatischer Chemosaturation trotz zielgerechter Antikoagulation: Ein Fallbericht

**DOI:** 10.1007/s00101-022-01175-y

**Published:** 2022-07-18

**Authors:** M. Kuhner, B. Tan, M. O. Fiedler, O. Biecker, B. Klein, D. H. Chang, M. A. Weigand, M. Dietrich

**Affiliations:** 1 Klinik für Anästhesiologie, Universitätsklinikum Heidelberg, Im Neuenheimer Feld 420, 69120 Heidelberg, Deutschland; 2 Abteilung für Kardiotechnik, Klinik für Herzchirurgie, Universitätsklinikum Heidelberg, Im Neuenheimer Feld 420, 69120 Heidelberg, Deutschland; 3 Klinik für Diagnostische und Interventionelle Radiologie, Universitätsklinikum Heidelberg, Im Neuenheimer Feld 420, 69120 Heidelberg, Deutschland

**Keywords:** Komplikationsmanagement, Gerinnung, Hämodynamik, Chemoperfusion, Melphalan, Complication management, Coagulation, Hemodynamic, Chemoperfusion, Melphalan

## Abstract

Die perkutane hepatische Chemosaturation ist eine Behandlungsoption bei nichtresektablen primären oder sekundären Lebertumoren. Dabei wird der Bereich der Lebervenenmündung der Vena cava inferior (VCI) mittels 2 Ballons von der Zirkulation isoliert, sodass die systemische Verteilung des über die Leberarterie applizierten Chemotherapeutikums Melphalan verhindert wird. Nach Passage der Leber und venöser Drainage aus der retrohepatischen VCI durchläuft das chemosaturierte Blut 2 parallel geschaltete extrakorporale Filter. Anschließend wird das gereinigte Blut jugulär rückgeführt. Das Verfahren geht oft mit einer ausgeprägten hämodynamischen Instabilität einher, deren Ursache nicht abschließend geklärt ist. Zusätzlich stellt das Gerinnungsmanagement eine Herausforderung dar. Die Autoren berichten von einem Fall, bei dem sich trotz ausreichender „activated clotting time“ (ACT) ein Thrombus im rückführenden Schenkel der extrakorporalen Zirkulation bildete. Gezielte Problemsuche und -lösung waren parallel zur hämodynamischen Stabilisierung und interdisziplinären Zusammenarbeit notwendig, um die Intervention erfolgreich durchzuführen und der Patientin eine sichere Therapie zukommen zu lassen.

## Abstract (en)

Percutaneous hepatic chemosaturation is a treatment option for unresectable primary or secondary liver tumors. In this procedure the part of the inferior vena cava (VCI) that collects blood from the hepatic veins is isolated using a double balloon catheter. Like this, systemic distribution of the chemotherapeutic agent melphalan which is administered via the hepatic artery can be prevented. After passage through the liver and drainage from the retrohepatic VCI, the chemosaturated blood passes through two extracorporeal filters. Subsequently, the filtered blood is returned via the jugular vein. The procedure is often accompanied by severe hemodynamic instability, the cause of which is still not completely understood. In addition, coagulation management of extracorporeal circulation is often challenging. The authors report a case in which a thrombus formed in the returning leg of the extracorporeal circulation despite sufficient activated clotting time (ACT). Targeted problem search and resolution were necessary simultaneously to hemodynamic stabilization and interdisciplinary collaboration to successfully perform the intervention and provide the patient with safe treatment.

## Einleitung

**Figure 1:**
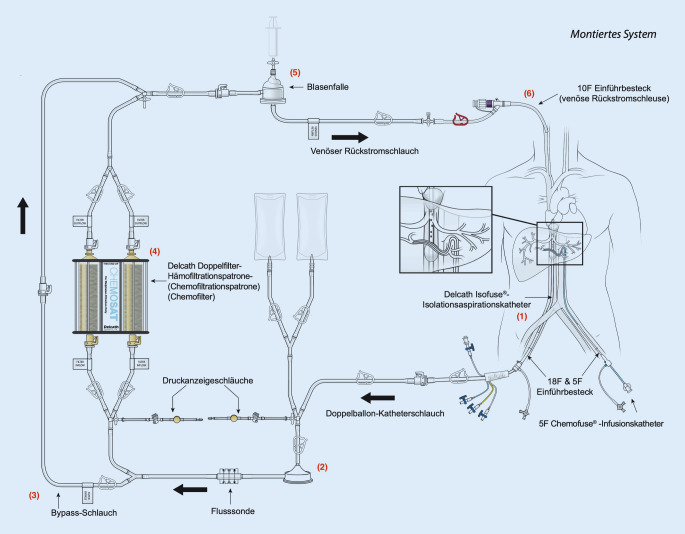

Fernmetastasen sind eine häufige Diagnose bei Aderhautmelanomen und werden in der Literatur mit bis zu 50 % beschrieben. Die Lokalisation ist meist hepatisch, was für die Patienten oft prognoseentscheidend ist [2, 5, 7, 10, 13]. Mit der hepatischen Melphalanchemosaturation existiert ein vergleichsweise neues Verfahren zur selektiven Hochdosistherapie von Lebertumoren, das in spezialisierten Zentren durchgeführt werden kann [1, 4, 6, 9]. Um die systemische Wirkung des über die Leberarterie applizierten Chemotherapeutikums Melphalan zu vermeiden, wird die Vena cava inferior (VCI) im Bereich der Lebervenenmündung mittels eines Doppelballonkatheters isoliert. Das dort über diesen Katheter drainierte, chemosaturierte Blut wird mittels diesem speziellen CHEMOSAT®-Katheter- und CHEMOSAT®-Filtersystems (Fa. Delcath Systems, Inc. New York, NY, USA) vom systemischen Kreislauf separiert ([3]; Abb. 1). Vor dem eigentlichen Eingriff erfolgen eine diagnostische Angiographie zum Ausschluss relevanter Gefäßvarianten und ggf. die protektive Embolisation ungünstiger extrahepatischer arterieller Gefäßkollateralen, um eine systemische Anreicherung des Zytostatikums zu vermeiden.

**Figure 2:**
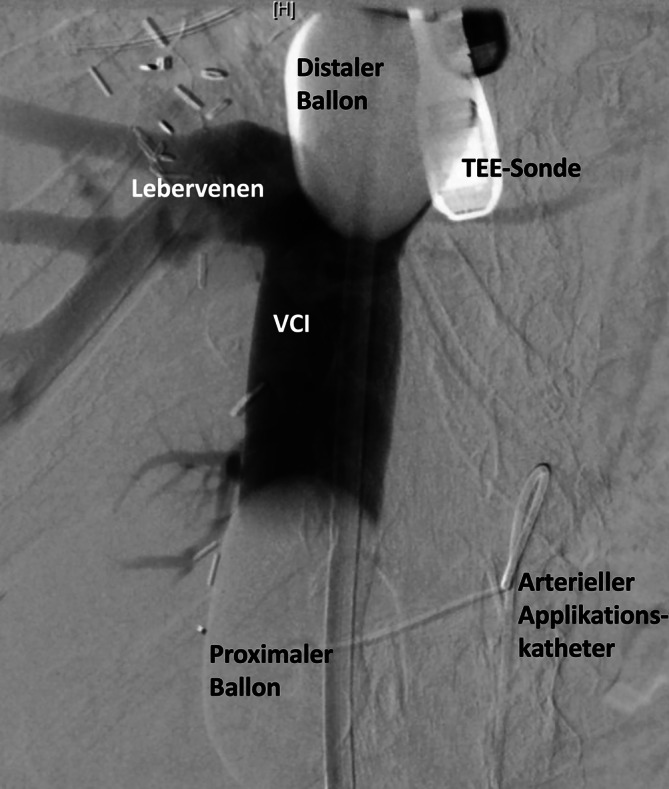

Im Rahmen der Chemosaturation wird Melphalan über einen in der A. hepatica platzierten Applikationskatheter gezielt der Leber zugeführt. Die systemische Verteilung wird durch einen in der inferioren V. cava retrohepatisch platzierten Doppelballonkatheter verhindert, über den das mit Melphalan gesättigte Blut abgesaugt und extrakorporal durch 2 parallel durchflossene Chemofilter geführt wird, in denen das Melphalan extrahiert wird (Abb. 2). Das so bereinigte Blut wird über eine in der V. jugularis interna platzierte Rückstromschleuse zurückgeführt. Anästhesiologisch handelt es sich um ein anspruchsvolles interventionelles Verfahren mit oft ausgeprägter hämodynamischer Instabilität sowie pro- und antikoagulatorischem Komplikationspotenzial. Herausforderungen liegen v. a. in der hämodynamischen Stabilisierung mittels differenzierter Vasopressor- und Flüssigkeitstherapie, um eine adäquate Gewebeperfusion aufrechtzuerhalten und damit ein postinterventionelles Organversagen sowie gleichzeitig eine exzessive Volumenüberladung zu verhindern. Zusätzlich steht das Gerinnungsmanagement der extrakorporalen Zirkulation (EKZ) während und nach dem Eingriff im Fokus, um sowohl thrombotische als auch Blutungskomplikationen zu vermeiden. Die hämodynamische Instabilität ist einerseits durch die kavale Ballonokklusion und andererseits während des Zuschaltens der Chemofilter beschrieben [8, 14, 15]. Für die Blutdruckabfälle während der Filterphase werden im Wesentlichen das Filtrieren von Katecholaminen sowie eine mögliche inflammatorische Reaktion an der Fremdoberfläche der Filter verantwortlich gemacht [11]. Die vollständigen pathophysiologischen Ursachen der hämodynamischen Veränderungen während der Chemosaturation und -filtration sind jedoch nicht ausreichend untersucht. Weitere Komplikationen betrafen v. a. Blutungen im Bereich der Gefäßzugänge mit häufig leichten, teilweise jedoch auch schwereren Verläufen mit Notwendigkeit der Reintubation und angiographischer Reintervention. Ein Anstieg der Transaminasen sowie Blutbildveränderungen wie Thrombozytopenie, Anämie und Neutropenie werden als häufige, teils transiente, oft jedoch auch transfusionspflichtige, laborchemische postinterventionelle Veränderungen beschrieben [7–9, 14]. Ein thrombotischer Verschluss ist eine gefürchtete Komplikation an der EKZ und wurde bereits in einer prospektiven Phase-II-Studie beschrieben[12]. Unserer Kenntnis nach existieren bisher jedoch noch keine publizierten detaillierten Fallberichte über eine periinterventionelle Koagelbildung in einem CHEMOSAT®-System trotz ACT-gesteuerter vermeintlich suffizienter Antikoagulation.

## Fallbericht

Wir berichten von einer 68-jährigen Patientin mit hepatisch metastasiertem Aderhautmelanom, die sich zur elektiven Durchführung der zweiten hepatischen Chemosaturation vorstellte. Die erste Chemosaturation war 6 Wochen zuvor durch unser anästhesiologisches und radiologisches Team komplikationslos durchgeführt worden.

Die Vorbereitung der Patientin verlief ereignislos. Mit Ausnahme von mehreren vorbeschriebenen Leberteilresektionen bei multipler hepatischer Metastasierung des Aderhautmelanoms sowie metastasensuspekten, jedoch größenkonstanten ossären Läsionen im Lendenwirbelkörper 3 und dem Beckenkamm bestanden keine signifikanten Vorerkrankungen. Insbesondere lagen keine kardiopulmonalen oder hämostaseologischen Begleiterkrankungen vor. Das Ausgangslabor war bis auf eine Hyperfibrinogenämie (6,54 g/l) und einer milden Thrombozytose (509/nl) unauffällig. Insbesondere die Antithrombin-III-Aktivität lag mit 108,5 % im Normbereich.

**Figure 3:**
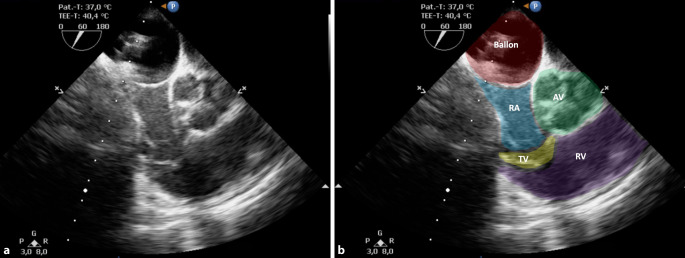

Am Interventionstag erfolgte die problemlose Narkoseeinleitung mittels Sufentanil, Propofol und Rocuronium. Die Aufrechterhaltung wurde mit Remifentanil und Sevofluran durchgeführt. Die Ausstattung der Patientin umfasste neben der Intubationsnarkose und den interventionsbedingt notwendigen Kathetern eine invasive Druckmessung über die A. radialis, einen zentralen Venenkatheter sowie die transösophageale Echokardiographie (TEE). Ein persistierendes Foramen ovale (PFO) wurde bereits im Rahmen der ersten Chemosaturation mittels TEE ausgeschlossen. Neben der Möglichkeit zum PFO-Ausschluss wird die TEE zur sicheren Platzierung des Doppelballonkatheters in ausreichender Entfernung zur Trikuspidalklappe verwendet (Abb. 3). Zudem kann sie zur Steuerung der hämodynamischen Therapie während Phasen ausgeprägter hämodynamischer Instabilität hilfreich sein.

Die Ausgangs-ACT (i-STAT Alinity Analyzer; Fa. Abbott Point of Care Diagnostics, Princeton, NY, USA) wurde mit 117 s bestimmt. Nach Gabe von 30.000 I.E. (440 I.E./kgKG) unfraktioniertem Heparin (UFH) i.v. lag diese mit 370 s noch unterhalb des Zielwertes von mindestens 400 s, sodass weitere 10.000 I.E. UFH (ca. 150 I.E./kgKG) appliziert wurden. Hierunter stieg die ACT auf 560 s an, sodass die Voraussetzungen für die EKZ gegeben waren. Diese wurde mit der Biomedicus Bio-Console 560 Centrifugal Pump (Fa. Medtronic, Minneapolis, MN, USA) durchgeführt. Das Priming war zuvor durch die Kollegen der Kardiotechnik mit Sterofundin ISO, 150 ml 5%iger Humanalbuminlösung sowie 5000 I.E. UFH durchgeführt worden.

Vor hepatischer Applikation des Melphalans wurden die beiden Ballons in der inferioren V. cava geblockt. Durch den reduzierten venösen Rückstrom kam es zu der erwarteten hämodynamischen Beeinträchtigung der Patientin. Echokardiographisch zeigte sich eine hyperdyname biventrikuläre Pumpunktion mit reduzierter ventrikulärer Füllung. Es zeigten sich keine Zeichen der Rechtsherzbelastung. Die Hämodynamik wurde differenziert mit kristalloidem Flüssigkeitsersatz und Vasopressoren behandelt. Primärer Vasopressor war Noradrenalin, welches zu diesem Zeitpunkt von 0,05 µg/kgKG und min bis auf 0,7 µg/kgKG und min gesteigert werden musste. Zusätzlich musste Vasopressin mit 0,03 I.E./min etabliert werden. Eine derartige hämodynamische Instabilität war für uns zu diesem frühen Zeitpunkt ungewöhnlich, da der Blutfluss der EKZ aktuell noch über den Bypass an den beiden Filtern vorbeigeführt wurde, die im Regelfall für den wesentlichen Anteil der hämodynamischen Instabilität verantwortlich gemacht und daher erst aufgeschaltet werden, wenn das Melphalan applikationsbereit ist. Eine weitere Auffälligkeit war ein nach kurzer Zeit hoher rückführender Druck bei gleichzeitig beinahe sistiertem Fluss der EKZ. Der hierdurch fehlende venöse Rückstrom war eine mögliche Erklärung für die ausgeprägte hämodynamische Reaktion. Die Auffälligkeiten von Hämodynamik und EKZ wurden umgehend im Team kommuniziert. So wurde der Entschluss gefasst, das Melphalan zunächst nicht zu applizieren und eine strukturierte Fehlersuche durchzuführen. Nach Verabreichung der Melphalanhochdosis kann das Verfahren erst nach einer 30-minütigen Auswaschphase über die Chemofilter unterbrochen werden, da andernfalls eine toxische Menge Melphalan in die systemische Zirkulation gelangen würde. Da die EKZ und somit auch die Chemofilter zu diesem Zeitpunkt jedoch nicht funktionsfähig waren, hätte eine Melphalanapplikation möglicherweise schwerwiegende Folgen. Der initiale Verdacht, einer Fehllage der rückführenden Schleuse, konnte durch die kontrastmittelgestützte röntgenologische Lagekontrolle sicher ausgeschlossen werden. Es zeigte sich nach Diskonnexion des rückführenden Schenkels von der Schleuse kein suffizienter Fluss, sodass das Problem auf den rückführenden Teil der EKZ distal der Pumpe eingegrenzt wurde. Um Zeit zur Identifizierung des Problems zu gewinnen, wurden die kavalen Ballons entblockt. Hierunter konnte die Noradrenalindosis reduziert und Vasopressin ausgeschlichen werden. Es bestand jedoch weiterhin ein Noradrenalinbedarf von 0,17 µg/kgKG und min. Währenddessen konnte durch den Kollegen der Kardiotechnik durch gezielte Suche ein Thrombus im arteriellen Filter des rückführenden Schenkels festgestellt werden. Dieser konnte zügig durch einen baugleichen Filter ersetzt werden, sodass ein kompletter Wechsel der EKZ und erneutes Priming, mit entsprechendem erheblichem Zeitverlust, vermieden werden konnten. Eine in der Zwischenzeit bestimmte ACT zeigte 460 s und lag somit noch im Zielbereich. Veranlasst durch die klinisch beobachtete Thrombenbildung wurden dennoch weitere 10.000 I.E. (ca. 150 I.E./kgKG) UFH verabreicht, mit dem Ziel, eine erhöhte ACT > 500 s zu erreichen. Diese lag nach dem Bolus bei 568 s. Nach Rekonnexion der EKZ und erneutem Blocken der beiden Ballons konnte nun ein suffizienter Fluss von 0,5 l/min mit akzeptablem Druck von 90 mm Hg im rückführenden Schenkel erreicht werden. Somit konnten die beiden Chemofilter zugeschaltet werden. Hierunter zeigte sich ein erneut deutlich ansteigender Katecholaminbedarf mit Dosierungen von Noradrenalin bis 1,2 µg/kgKG und min und Vasopressin bis 0,06 IU/min. Unter dieser Therapie zeigte sich eine stabilisierte Hämodynamik für die restliche Intervention. Mit einem weiteren UFH-Bolus von 10.000 I.E. (ca. 150 I.E./kgKG) blieb die ACT weiterhin im Zielbereich, und es zeigten sich keine weiteren thrombotischen Ereignisse, sodass die Chemosaturation erfolgreich durchgeführt werden konnte. Mit dem Ende der extrakorporalen Filtration und dem Entblocken des Doppelballonkatheters konnte die katecholaminerge Unterstützung erwartungsgemäß deutlich reduziert und zur unmittelbar postinterventionellen Extubation komplett beendet werden. Nach Beendigung der EKZ wurde die Heparinwirkung mittels 22.000 I.E. Protamin (ca. 325 I.E./kgKG, entsprechend ca. 35 % der kumulativen UFH-Dosis) aufgehoben, wodurch sich eine postinterventionelle ACT von 133 s ergab. Gesamtbilanz bis zu diesem Zeitpunkt waren eine Einfuhr von 6000 ml Kristalloiden, 200 ml 20 %iger Humanalbuminlösung (HA), 150 ml 5 %iger HA durch das Priming der EKZ sowie 300 ml Erythrozytenkonzentrat (EK). Durch Punktion, Diskonnexionen, Spülen und Systemteilwechsel ergaben sich ein Blutverlust von ca. 500 ml sowie eine Ausscheidung von 750 ml Urin über 6 h. Ein Transfusionsbedarf zeigt sich nach Erfahrung der Autoren hier nicht regelhaft. In diesem Fall zeigte sich durch den genannten Blutverlust sowie durch Dilution allerdings ein Hb-Abfall bis 6,8 g/dl, wodurch die Indikation zur EK-Transfusion gestellt wurde. Die Patientin konnte unmittelbar postinterventionell wach, spontan atmend, kardiozirkulatorisch stabil und ohne erkennbares neurologisches Defizit auf der Intensivstation übergeben werden.

## Diskussion

Thrombotische Ereignisse sind bedrohliche Komplikationen extrakorporaler Verfahren. Zwar war in diesem Fall nicht die Kreislauf- und/oder Lungenfunktion, wie beim „extracorporeal life support“ (ECLS) oder der extrakorporalen Membranoxygenierung (ECMO), von der EKZ abhängig; nichtsdestotrotz ergaben sich aus der Komplikation eine erhebliche hämodynamische Problematik und Bedrohung der Patientin. Außerdem musste der Abbruch des Verfahrens in Betracht gezogen werden. Wäre die Komplikation nach Gabe des Melphalans aufgetreten, hätte dies möglicherweise lebensbedrohliche Folgen durch die systemische Applikation und Toxizität des hochdosierten Chemotherapeutikums gehabt. Die interdisziplinäre Diskussion im Rahmen der Komplikation wurde hier lösungsorientiert geführt, um das Problem zu identifizieren. Nachdem dies erfolgt war, stellte sich die Frage, ob die EKZ ausreichend zeitnah wieder suffizient reetabliert werden konnte und die Verzögerung für die Patientin bei bestehender ausgeprägter hämodynamischer Instabilität sowie die zu erwartende weitere Destabilisierung durch das Aufschalten der Chemofilter medizinisch vertreten werden konnten. Es musste in Betracht gezogen werden, dass weitere thrombotische Ereignisse mit oben beschriebenen katastrophalen Folgen möglich gewesen wären. Als sich abzeichnete, dass die EKZ zeitnah reetabliert werden konnte und der Patientenzustand auf oben genanntem Niveau stabil blieb, fiel die interdisziplinäre Entscheidung, das Verfahren unter höheren ACT-Zielwerten von 500 s und engmaschiger Kontrolle alle 15–20 min fortzuführen. Die genaue Ursache der Thrombose konnte letztlich nicht sicher identifiziert werden. Ausgeschlossen werden konnten Fehlmessungen der ACT, z. B. durch Abnahme aus dem Heparinlumen, sowie geknickte Leitungen, die durch eine Stase die Gerinnselbildung auslösen könnten. Auch eine verlängerte Zeit vom Einbringen des Doppelballonkatheters bis zum Start der EKZ kann durch Stase und Gerinnungsaktivierung durch Fremdoberflächenkontakt eine mögliche Quelle von Thromben sein. Dies führte in der Folge des beschriebenen Falls zur Änderung des Arbeitsablaufs, sodass fortan der Doppelballonkatheter bereits an die EKZ konnektiert und retrograd „geprimt“ eingeführt wird, um die Zeit bis zum Start der EKZ zu minimieren. Die vorbestehende Hyperfibrinogenämie und milde Thrombozytose könnten ebenfalls zur Thrombusformation beigetragen haben und könnten für zukünftige Prozeduren eine großzügigere Antikoagulation mit höherem ACT-Ziel erwägen lassen.

## Fazit für die Praxis

Die Chemosaturation ist eine auch aus anästhesiologischer Sicht herausfordernde Intervention mit dem Fokus auf:

- differenzierter hämodynamischer Stabilisierung,
- sorgfältiger Balance pro-/antikoagulatorischer Maßnahmen.

Die sichere Durchführung erfordert neben anästhesiologischen Kenntnissen und Fertigkeiten auch detailliertes Wissen über die Intervention an sich sowie eine lösungs- und patientenorientierte Zusammenarbeit im multiprofessionellen Team.
